# Study on cyanidin metabolism in petals of pink-flowered strawberry based on transcriptome sequencing and metabolite analysis

**DOI:** 10.1186/s12870-019-2048-8

**Published:** 2019-10-14

**Authors:** Li Xue, Jian Wang, Jun Zhao, Yang Zheng, Hai-Feng Wang, Xue Wu, Cheng Xian, Jia-Jun Lei, Chuan-Fei Zhong, Yun-Tao Zhang

**Affiliations:** 1 College of Horticulture, Shenyang Agricultural University, Shenyang, 110866 Liaoning China; 2 Beijing Academy of Agriculture and Forestry Sciences, Beijing, 100093 China

**Keywords:** Pink-flowered strawberry, Flower color, Pigmentation, Cyanidin, Transcriptome analysis, Anthocyanin biosynthesis pathway

## Abstract

**Background:**

Pink-flowered strawberry is a promising new ornamental flower derived from intergeneric hybridization (*Fragaria* × *Potentilla*) with bright color, a prolonged flowering period and edible fruits. Its flower color ranges from light pink to red. Pigment compounds accumulated in its fruits were the same as in cultivated strawberry fruits, but different from that in its flowers. However, the transcriptional events underlying the anthocyanin biosynthetic pathway have not been fully characterized in petal coloration. To gain insights into the regulatory networks related to anthocyanin biosynthesis and identify the key genes, we performed an integrated analysis of the transcriptome and metabolome in petals of pink-flowered strawberry.

**Results:**

The main pigments of red and dark pink petals were anthocyanins, among which cyanidins were the main compound. There were no anthocyanins detected in the white-flowered hybrids. A total of 50,285 non-redundant unigenes were obtained from the transcriptome databases involved in red petals of pink-flowered strawberry cultivar Sijihong at three development stages. Amongst the unigenes found to show significant differential expression, 57 were associated with anthocyanin or other flavonoid biosynthesis, in which they were regulated by 241 differentially expressed members of transcription factor families, such as 40 MYBs, 47 bHLHs, and 41 NACs. Based on a comprehensive analysis relating pigment compounds to gene expression profiles, the mechanism of flower coloration was examined in pink-flowered strawberry. A new hypothesis was proposed to explain the lack of color phenotype of the white-flowered strawberry hybrids based on the transcriptome analysis. The expression patterns of *FpDFR* and *FpANS* genes corresponded to the accumulation patterns of cyanidin contents in pink-flowered strawberry hybrids with different shades of pink. Moreover, *FpANS*, *FpBZ1* and *FpUGT75C1* genes were the major factors that led to the absence of anthocyanins in the white petals of pink-flowered strawberry hybrids. Meanwhile, the competitive effect of *FpFLS* and *FpDFR* genes might further inhibit anthocyanin synthesis.

**Conclusions:**

The data presented herein are important for understanding the molecular mechanisms underlying the petal pigmentation and will be powerful for integrating novel potential target genes to breed valuable pink-flowered strawberry cultivars.

## Background

Strawberry is known for its unique aroma, sweet taste, and rich nutrients [1, 2]. As one of the highly valued berry crops, commercial, ornamental pink-flowered strawberry (PFS) cultivars have been developed, from the cross between white-flowered strawberry (*Fragaria* × *ananassa* Duch.) and purple-flowered marsh cinquefoil (*Potentilla palustis* (L.) Scop.) [3]. The PFS is popular for its bright color, prolonged flowering period and edible fruit [4]. It can be used as ground cover, a pot plant, or in a hanging basket [5]. Several ornamental PFS cultivars have been released worldwide in the last two decades, such as Pikan, Viva Rosa, Tarpan, Toscana, Sijihong and Xiaotaohong [6]. However, in China, the breeding of PFS has been carried out only recently.

Flower color is one of the most important characteristics of ornamental plants, which is affected by the different kinds of plant pigments, such as flavonoids, carotenoids and betalains [7, 8]. Anthocyanins, a group of flavonoids, are the principal flower pigments, imparting color to flowers, ranging from light pink to violet. Varying shades of ornamental peach flowers has been attributed to cyanidins (Cys), whose content considerably varied in red, pink, and variegated petals [9]. Anthocyanins are synthesized via the ubiquitous and well-described secondary metabolic pathway in plants, such as *Antirrhinum majus*, *Gerbera hybrida*, *Prunus persica*, and *Triticum aestivum* [10–13]. Nevertheless, the molecular mechanism regulating anthocyanin synthesis in different plant species has not been identified, because of the structural diversity of anthocyanins [14].

Considerable progress has been made over the past decades to characterize the diverse pigment compounds among different strawberry fruits and to uncover the genes and mechanisms involved in the accumulation of different pigments via biosynthetic pathways [15–18]. The main anthocyanins in strawberry fruits are derived from pelargonidin (Pg) and Cy aglycones. The major pigment in cultivated strawberries has been identified as pelargonidin-3-glucoside, which confers a bright red color to the receptacle [19, 20]. Interestingly, the main type of anthocyanin in PFS petals, cyanidin-3-O-glucoside (Cy3G), is different from that accumulated in strawberry fruits [21]. The mechanism underlying the anthocyanin biosynthetic pathway (ABP) in strawberry fruit has been characterized, but no information is available on flower coloration in PFS.

The mechanism involved in flower color variation is complex, because the ABP is tightly regulated by multiple exogenous and endogenous factors through regulatory networks, including several transcription factors [22, 23]. Once a candidate gene is identified with either sequence or expression differences that correlate with flower color, subsequent biochemical analysis of the petal can be used to evaluate the flavonoid composition by high-performance liquid chromatography coupled with mass spectrometry (HPLC-MS). In particular, transcript and metabolite datasets have been combined via correlation and clustering analyses and further represented as connection networks between genes and metabolites in several plants, including tobacco [24], soybean [25] and kale [26]. Through a combination of chemical analysis and transcriptome analysis, the major metabolic pathways related to *Muscari* flower pigmentation have been deduced and the candidate genes targeting the loss of pigments in the plants have been examined [27]. In the present study, we explored the regulatory networks of the ABP in the petals of PFS at the transcriptome and metabolome levels. The RNA-Seq for PFS flower color was performed using the Illumina sequencing technique and HPLC-MS was used to identify and quantify the flavonoid composition. Connection networks were mapped between metabolites and transcripts to highlight the regulatory genes associated with anthocyanin metabolites. Our study will provide new insights for the subsequent study on complex physiological processes and molecular mechanisms associated with the biosynthesis and regulation of anthocyanins in the flowers of PFS.

## Results

### Major classes of coloration compounds in PFS petals

**Fig. 1:**
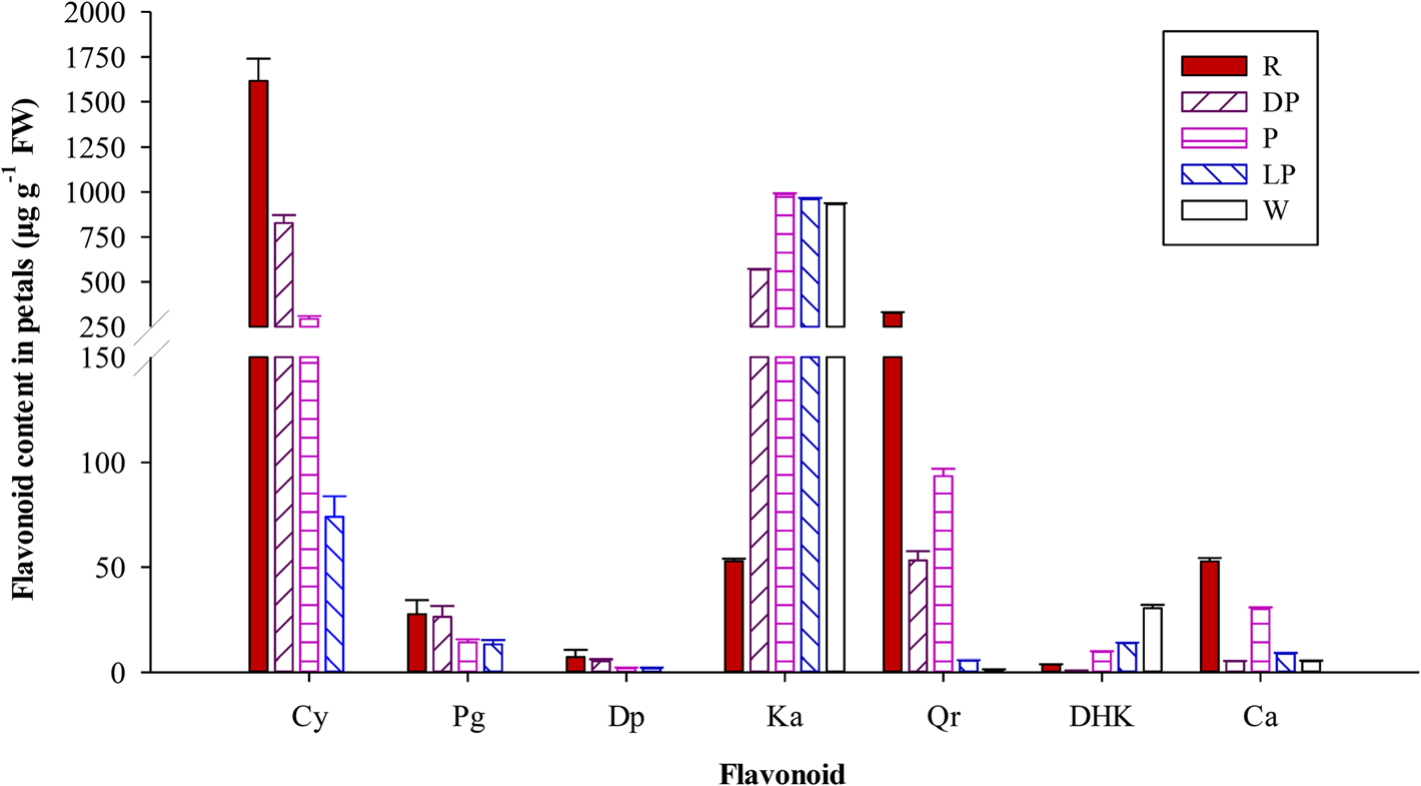
Flavonoid compositions of different color petals in pink-flowered strawberry. Cy. Cyanidin, Pg. Pelargonin, Dp. Delphinidin, Ka. Kaempferol, Qr. Quercetin, DHK. Dihydrokaempferol, Ca. Catechin. Different flower colors: R. Red, DP. Dark pink, P. Pink, LP. Light pink, W. White

The branching products of the flavonoid biosynthesis pathway (FBP) in different-colored petals of PFS were determined to examine the metabolic patterns. The results showed that the main coloration compounds of red and dark pink petals were anthocyanins, among which accounted for 79.08 and 57.78% of the total flavonoid (TFL) content in the red and dark pink petal. As the flower color deepened, the anthocyanin content increased, and the red petal showed the highest TFL content, followed by the dark pink, pink, light pink and white petals, in which the anthocyanin content was 1651.38, 858.73, 310.51, 89.05, and 0.00 μg·g^− 1^ (Table 1). The PFS contained three anthocyanin compounds, namely Cy, Pg and delphinidins (Del), among which Cy was the major anthocyanin. The red petals presented the highest Cy content, accounting for 77.43% of the TFL content, which was 1.95, 5.49, and 21.83 times higher than that in the dark pink, pink and light pink petals, respectively (Table 1 and Fig. 1). No anthocyanin was detected in white-flowered hybrids. The content of kaempferol (Ka) in the pink, light pink, and white petals was almost the same, which was 18.74 times higher than that in the red petal. The white petal presented the highest dihydrokaempferol (DHK) content, accounting for approximately 3.13% of the TFL content, followed by the light pink, whereas the red and dark pink petals presented the lowest content. The pink-flowered strawberry cultivar Sijihong showed dark red flowers. In its different developmental stages, the big bud stage (D) had the highest total anthocyanin (TA) content with 1670.02 μg·g^− 1^, followed by half opened stage (B) with 1613.09 μg·g^− 1^, and no anthocyanin was detected in the young bud stage (L) (Additional file 1: Table S1).

**Table 1:** The contents of flavonoids in petals of pink-flowered strawberry hybrids with different color

Flavonoid types		Red		Dark pink		Pink		Light pink		White	
		Contents (μg·g^− 1^ fw)	Percent(%)	Contents (μg·g^− 1^ fw)	Percent(%)	Contents (μg·g^− 1^ fw)	Percent(%)	Contents (μg·g^− 1^ fw)	Percent(%)	Contents (μg·g^− 1^ fw)	Percent(%)
Anthocyanidins	Cyanidins	1616.76 ± 123.2	77.43	827.29 ± 44.44	55.66	294.43 ± 14.67	20.56	74.07 ± 9.73	6.87	–	–
	Pelargonins	27.53 ± 6.69	1.31	26.24 ± 5.16	1.77	14.19 ± 1.29	0.99	13.13 ± 2.01	1.22	–	–
	Delphinidins	7.09 ± 3.32	0.34	5.2 ± 0.9	0.35	1.89 ± 0.38	0.13	1.85 ± 0.34	0.17	–	–
Flavanones	Naringenins	–	–	–	–	–	–	–	–	–	–
Flavonols	Myricetins	–	–	–	–	–	–	–	–	–	–
	Quercetins	327.35 ± 3.61	15.68	53.15 ± 4.43	3.58	93.4 ± 3.56	6.52	5.57 ± 0.13	0.52	1.21 ± 0.19	0.12
	Kaempferols	52.76 ± 1.18	2.53	568.42 ± 3.64	38.24	988.74 ± 3.83	68.99	960.58 ± 6.09	89.15	932.81 ± 5.09	96.21
	Dihydromyricetins	–	–	–	–	–	–	–	–	–	–
	Dihydroquercetins	–	–	–	–	–	–	–	–	–	–
	Dihydrokaempferols	3.63 ± 0.13	0.17	0.8 ± 0.08	0.05	9.55 ± 0.3	0.67	13.6 ± 0.26	1.26	30.32 ± 1.58	3.13
Procyanidines	Catechins	52.67 ± 1.67	2.54	5.16 ± 0.3	0.35	29.99 ± 0.77	2.26	8.7 ± 0.35	0.81	5.25 ± 0.27	0.54
	Epicatechins	–	–	–	–	–	–	–	–	–	–
Total		2087.79 ± 5.88	100.00	1486.26 ± 1.04	100.00	1432.18 ± 8.46	100.00	1077.49 ± 6.82	100.00	969.60 ± 4.84	100.00

- means failure to be detected

### RNA-Seq and assembly

The original data obtained by sequencing with Illumina Hiseq 4000 were transformed into raw reads via base calling. The total base number obtained from nine libraries was 70.6 G and the total number of nucleotides was greater than 5.5 Gb in each sample (Additional file 2: Table S2). The Q20 ratio of each sample was greater than 98% and GC content was relatively consistent, around 46%. A total of 164,727 transcripts and 50,285 non-redundant unigenes were obtained from nine databases, respectively. The average length of unigenes was 548 bp and length of N50 was 1394 bp (Additional file 3: Table S3). Thus, this sequencing data quality was high and could meet the requirements of the subsequent analysis. There were 23,669 unigenes with length of 200–500 bp accounting for 47.07%, 11,143 unigenes of length 500–1000 bp accounting for 22.16%, 10,846 unigenes of length of 1000–2000 bp accounting for 21.57%, and 4627 of length more than 2000 bp accounting for 9.2% (Additional file 4: Table S4).

### Identification of differentially expressed genes (DEGs) in PFS

To gain a whole view of gene expression pattern across nine petal transcriptomes, a Pearson’s distance correlation matrix was generated using the normalized TPM values (Additional file 5: Figure S1A). All biological replicates were clustered together with almost identical expression profiles (R > 0.9), indicating the reliability of sample collection and analytical procedure. A total of 13,815 genes were differentially expressed among the three samples, and 1426 genes were differentially expressed in all the three samples (Additional file 5: Figure S1B). There were 4354 DEGs in PF_Z vs PF_L, among which 1731 genes were downregulated and 2623 genes were upregulated. There were at most 11,252 DEGs between the PF_D and PF_L samples, whose flower color varied significantly, and 6376 genes were downregulated and 4876 genes were upregulated. There were 8919 DEGs between PF_D and PF_Z, among which 5586 genes were downregulated and 3333 genes were up-regulated (Additional file 5: Figure S1C).

### Functional annotation of unigenes

Based on the Nr annotation results, the plant species with the highest frequency in the annotation results of the PFS database (top 8 places) were obtained (Additional file 6: Figure S2), and *F. vesca* presented the highest frequency in the annotation results with a total of 27,709 comments, accounting for 89.46% of all the sequences. That was followed by other genera of the Rosaceae, such as *Prunus persica* (1.9%), *Prunus mume* (1.03%), *Malus domestica* (0.92%), and *Pyrus bretschneideri* (0.68%).

**Fig. 2:**
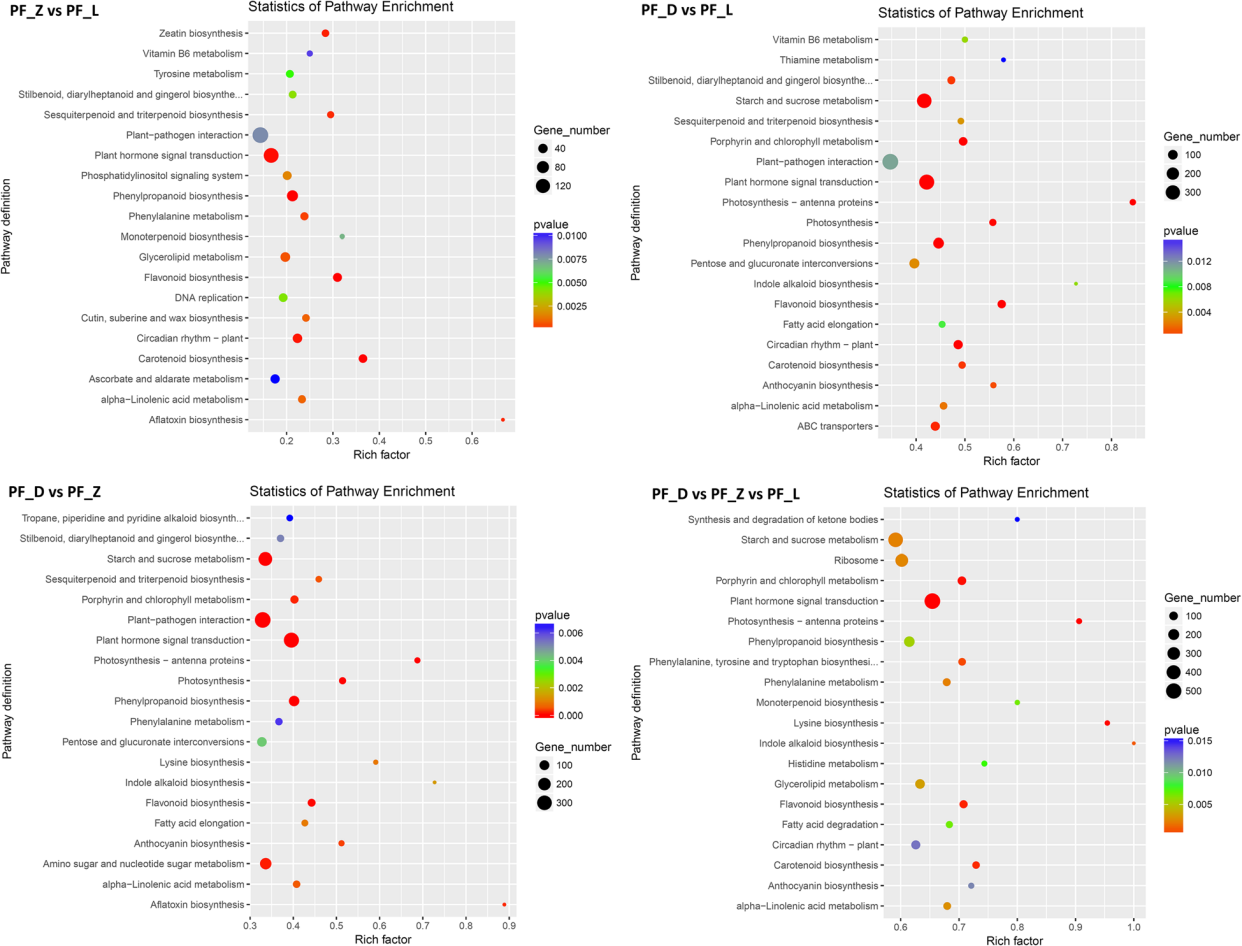
Top 20 KEGG pathways with the most significant enrichment during petal development. L. Young bud stage, Z. Beginning coloration stage, D. Big bud stage

A total of 24,311 (48.35%) unigenes were mapped to the Gene Ontology (GO) terms in the cellular component, molecular function, and biological process categories and were further classified into 50 terms. In the cellular component category, the DEGs in PF_D vs, PF_Z vs, PF_L, which indicated the union of all differentially expressed genes, were mainly related to the nucleus, cytoplasm, and integral membrane component. In the molecular function category, most of the DEGs were enriched for molecular function, protein binding, and ATP binding. In the biological process, regulation of transcription, DNA template and protein phosphorylation were mainly involved (Additional file 7: Figure S3). To identify the metabolic pathways during petal development, 7032 DEGs were mapped to the Kyoto Encyclopedia of Genes and Genomes (KEGG) database, and the top 20 KEGG pathways significantly enriched were shown in Fig. 2. Among these pathways, 534 DEGs in the PF_D vs, PF_Z vs, PF_L group were enriched in plant hormone signal transduction pathways, suggesting that hormones might play crucial roles in the regulation of flower development and coloration. Furthermore, numerous genes were involved in the pathways related to phenylpropanoid biosynthesis, starch and sucrose metabolism, and amino sugar and nucleotide sugar metabolism. Anthocyanins and flavonols were the most important pigments in flower coloration in PFS. Therefore, we focused on the FBP and ABP in this study.

### DEGs associated with ABP

To identify genes related to flower coloration, the key genes of all secondary metabolic pathways involved in the ABP and FBP were determined from the transcriptome database. Fifty-seven DEGs were identified to be associated with anthocyanin biosynthesis, anthocyanin modification, flavone and flavonol biosynthesis, and flavanone biosynthesis (Table 2). To gain insights into the functional roles of genes related to flower coloration during petal development, a heatmap was plotted using the Z-score normalized to TPM values (Additional file 8: Figure S4). All these DEGs exhibited differential expressions in different flower development stages. In the early anthocyanin biosynthesis, three DEGs encoding chalcone synthase (CHS) were annotated, in which all of them were upregulated in PF_D vs PF_L; two DEGs encoding chalcone isomerase (CHI), five DEGs encoding flavanone 3-hydroxylase (F3H), three DEGs encoding flavonoid 3′-hydroxylase (F3’H), and four DEGs encoding flavonoid 3′, 5′-hydroxylase (F3’5’H) were annotated. In later biosynthesis, four DEGs encoding dihydroflavonol 4-reductase (DFR) and two DEGs encoding anthocyanidin synthase (ANS) were all predominantly expressed in PF_D, suggesting a key role in the regulation of petal coloration in PFS; eight DEGs encoding anthocyanidin 3-O-glucosyltransfersae (BZ1) were annotated. Several DEGs associated with anthocyanin modification were identified, such as anthocyanidin 5, 3-O-glucosyltransferas (GT1), anthocyanin 3-O-glucoside 5-O-glucosyltransferase (UFGT75C1), anthocyanidin 3-O-glucoside 2″‘-O-xylosyltransferase (UGT79B1), and cyanidin-3-O-glucoside 2″-O-glucuronosyltransferase (UGAT). In addition, five DEGs encoding flavonol synthase (FLS) were also identified.

**Table 2:** Candidate genes related to flower pigmentation of pink-flowered strawberry

Function	Genes	Enzymes	KO id (EC-No.)	All^a^	Up-regulated in PF_L^b^	Up-regulated in PF_D^c^
Anthocyanin biosynthesis	CHS	Chalcone synthase	K00660 (2.3.1.74)	3	0	3
	CHI	Chalcone isomerase	K01859 (5.5.1.6)	2	0	0
	F3H	Flavanone 3-hydroxylase	K00457 (1.14.11.9)	5	0	3
	F3’H	Flavonoid 3′-hydroxylase	K05280 (1.14.13.21)	3	2	1
	F3’5’H	Flavonoid 3′, 5′-hydroxylase	K13083 (1.14.13.88)	4	3	0
	DFR	Dihydroflavonol 4-reductase	K13082 (1.1.1.219)	4	0	4
	ANS	Anthocyanidin synthase	K05277 (1.14.11.19)	2	0	2
	BZ1	Anthocyanidin 3-O-glucosyltransfersae	K12930 (2.4.1.115)	8	4	1
Anthocyanin modification	GT1	Anthocyanidin 5, 3-O-glucosyltransferase	K12938 (2.4.1.-)	2	1	0
	UFGT75C1	Anthocyanin 3-O-glucoside 5-O-glucosyltransferase	K12338 (2.4.1.298)	5	1	3
	UGT79B1	Anthocyanidin 3-O-glucoside 2″‘-O-xylosyltransferase	K17193 (2.4.2.51)	3	1	0
	UGAT	Cyanidin-3-O-glucoside 2″-O-glucuronosyltransferase	K12937 (2.4.1.254)	5	3	1
Flavone and flavonol biosynthesis	FLS	Flavonol synthase	K05278 (1.14.11.23)	5	2	1
Flavanone biosynthesis	ANR	Anthocyanidin reductase	K08695 (1.3.1.77)	2	0	1
	LAR	Leucoanthocyanidin reductase	K13081 (1.17.1.3)	4	1	0

All^a^ indicates the total number of DEGs; PF_L^b^ indicates the number of DEGs up-regulated in PF_L; PF_D^c^ indicates the number of DEGs up-regulated in PF_D

**Fig. 3:**
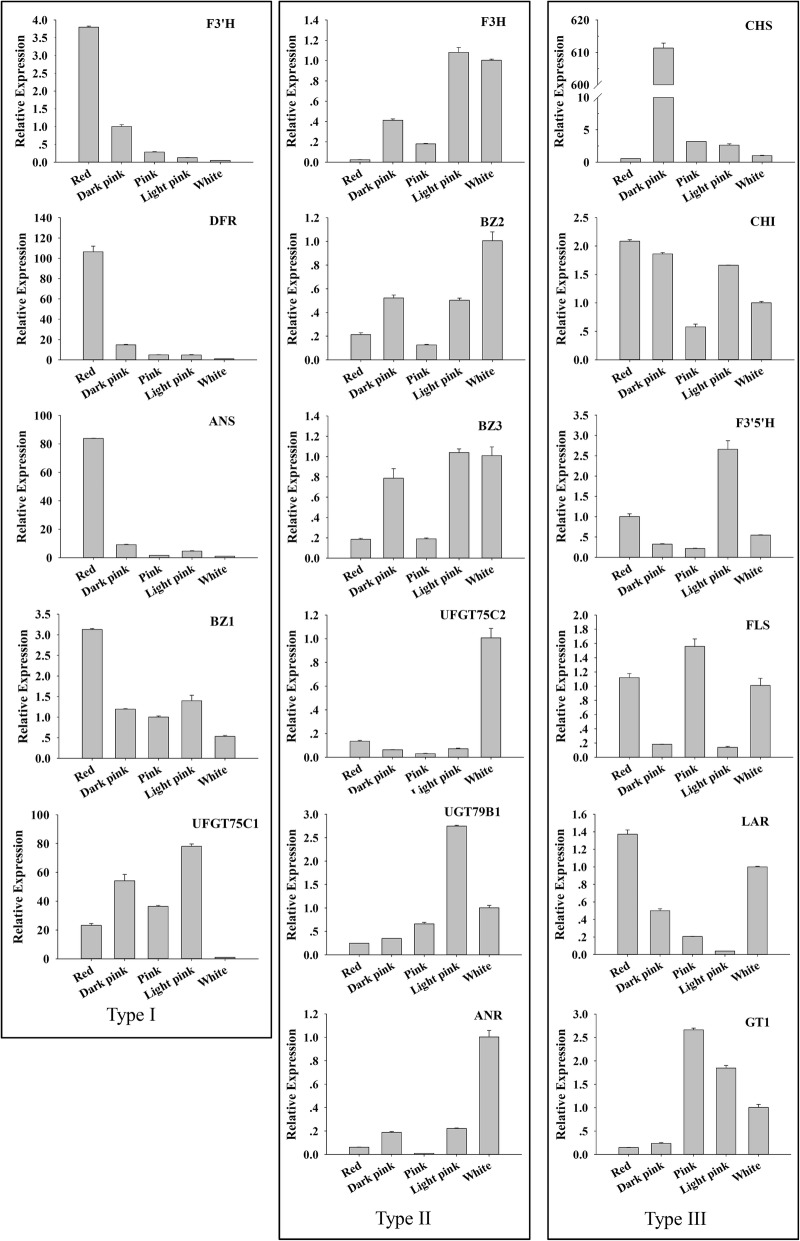
Expression levels of anthocyanin pathway genes in pink-flowered strawberry hybrids with different shades of pink and white. All reactions of qRT-PCR were repeated three times for each sample, and vertical bars indicated standard errors

To validate the reliability of the expression profiling obtained by RNA-seq, seventeen genes related to flower coloration were selected for qRT-PCR analysis. For all these genes, the results of qRT-PCR exhibited almost identical expression patterns as compared to the transcriptome sequencing data (Additional file 9: Figure S5). The expression of 17 key DEGs was detected in the petals of five different colors, namely white, light pink, pink, dark pink and red (Fig. 3). According to the qRT-PCR results of 17 genes, their expression patterns could be divided into three groups. The first type included the *FpF3’H*, *FpANS*, *FpDFR*, *FpBZ1* and *FpUGT75C1* genes, and their expression was the lowest in white flowers; among them the *FpF3’H*, *FpANS*, *FpDFR* and *FpBZ1* genes showed a similar trend between gene expression and anthocyanin accumulation. Although the *FpUGT75C1* gene expression was the lowest in white flowers, its expression pattern was not directly related to other colors. The second type included the *FpF3H*, *FpBZ2*, *FpBZ3*, *FpANR*, *FpUGT75C2*, and *FpUGT79B1* genes. Their expression in white flowers was higher than that in red flowers. The third type included the *FpCHS*, *FpCHI*, *FpF3’5’H*, *FpLAR*, *FpFLS*, and *FpGT1*, and their expression was inconsistent in different flower colors. For example, the *FpCHS* gene had differential expression patterns in different flower colors. The expression of the *FpCHS* gene was the lowest in red flowers, which may be caused by different genetic background.

### Identification of transcription factors (TFs) related with ABP

**Fig. 4:**
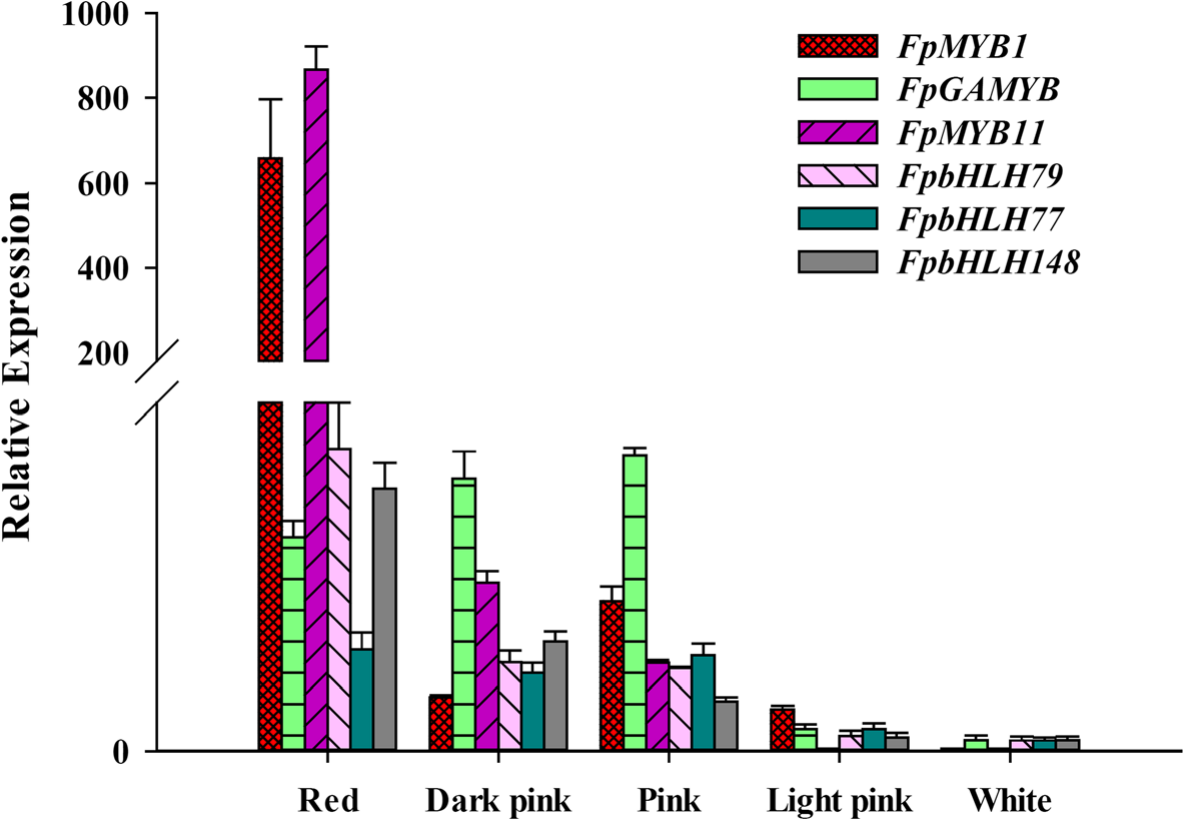
Expression levels of transcription factors in pink-flowered strawberry hybrids with different shades of pink and white (*FpMYB1*, *FpGAMYB*, *FpMYB11*, *FpbHLH79*, *FpbHLH77*, and *FpbHLH148*). All reactions of qRT-PCR were repeated three times for each sample, and vertical bars indicated standard errors

Several TFs have been reported to play important roles in ABP. In our study, 241 putative TF-encoding genes belonging to 8 TF families were found to be differentially expressed in the PF_D vs, PF_Z vs, PF_L, such as MYB (40 genes), bHLH (47 genes), NAC (41 genes), and WRKY families (47 genes). The numbers of differential expressed TFs related with ABP in the three development stages were showed in Additional file 10: Table S5. To screen key TFs for anthocyanin synthesis, the expression of all those TF genes was showed in a heatmap (Additional file 11: Figure S6). Expression levels of those TFs selected were different in development stages. Three MYB and bHLH DEGs (*FpMYB1*, *FpGAMYB*, *FpMYB11*, *FpbHLH79*, *FpbHLH77*, and *FpbHLH148*) were predominantly expressed in PF_D and selected for qRT-PCR analysis. They had higher expression in red-flowered strawberry than in white-flowered strawberry (Fig. 4).

## Discussion

### Candidates responsible for loss of red color in white-flowered hybrids

To examine why the white petals of PFS hybrids could not accumulate anthocyanins at the transcriptome level, the expression pattern of all key structural genes of FBP and the content of pigment compounds were compared. Based on transcriptome sequencing and qRT-PCR results, the expression of the *FpF3’H*, *FpDFR*, *FpANS*, *FpBZ1*, and *FpUGT75C1* genes was significantly increased in the red petals, as the flower color deepened (Fig. 3). These genes and their corresponding catalytic reactions might be key genes and speed-limiting steps leading to the loss of anthocyanins in the white petals.

**Fig. 5:**
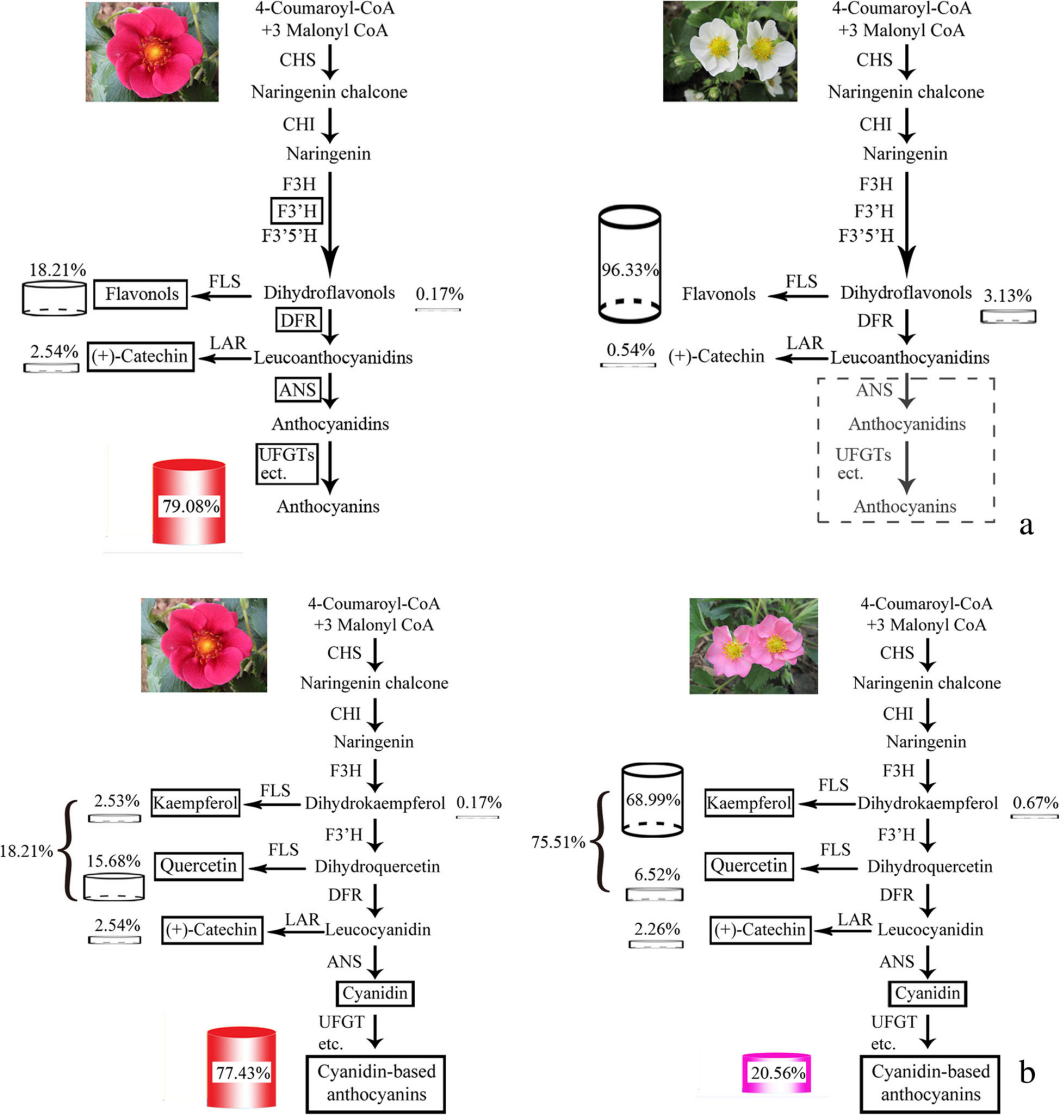
Two models for the process of anthocyanins elimination in white-flowered strawberry (**a**), and metabolic process in red-flowered and pink-flowered strawberry (**b**), respectively. CHS. Chalcone synthase, CHI. Chalcone isomerase, F3H. Flavanone 3-hydroxylase, F3’H. Flavonoid 3′-hydroxylase, F3’5’H. Flavonoid 3′,5′-hydroxylase, DFR. Dihydroflavonol 4-reductase, ANS. Anthocyanidin synthase, UFGT. UDP-glucose: Flavonoid 3-O-glucosyltransferase, FLS. Flavonol synthase, LAR. Leucoanthocyandin reductase

Firstly, although the *FpF3’H* gene can effectively restrict the Cy metabolism branch, catechin (Ca) was found in the white petals, suggesting that the white petals were due to the lack of anthocyanin metabolism downstream genes. Meanwhile, the content of DHK in the white petals was 8.32 times higher than that in the red petals, indicating that the ABP in the white petals was restrained and some amount of DHK was accumulated (Table 1). Secondly, the *FpANS* gene also played a key role in the ABP, which could catalyze the synthesis of colorless anthocyanins into colored anthocyanins. If the white petals were blocked in that position, the colored anthocyanins would disappear (Fig. 5a). No anthocyanin was detected in the white petals in this experiment, and therefore, the *FpANS* gene might be the key factor in the inability to accumulate anthocyanins in the white petals. As evidenced from the qRT-PCR results, the *FpANS* gene expression in the red petals was 100 times higher than that in the white petals, and in the big bud developmental stage, its expression was even up to 1000 times higher (unpublished). Thirdly, glycosyltransferase was directly related to the final product, and the synthesized anthocyanidins would be transformed into stable anthocyanins under its action (Fig. 5a). In this study, *FpBZ1* and *FpUGT75C1* were significantly upregulated in the red petals, and therefore, they might also be key factors in the inability to accumulate anthocyanins in the white petals. In addition, from the perspective of the branch products of ABP, Cy cannot be accumulated due to the influence of other substrate competition (the flavone and flavonol metabolic pathways, and procyanidins metabolic pathways). The *FpFLS* gene along with the *FpDFR* gene could compete for a substrate, and under the action of the FpFLS protein forming a colorless Kas and quercetins (Qrs). The flavonol content in the white petals in this study was 2.46 times higher than that in the red petals. It was also verified that the proportion of dihydroflavonol converted into flavonols than into anthocyanins was higher in white flowers. Therefore, it can be inferred that the *FpANS*, *FpBZ1*, and *FpUGT75C1* genes are the key factors that lead to failure to in the accumulation of anthocyanins in white-flowered hybrids. Meanwhile, the competitive effect of *FpFLS* and *FpDFR* genes might further inhibit anthocyanin synthesis.

### Flower coloration with different shades of pink

PFS hybrids presented varying shades of pink, ranging from light pink to red. To examine the reasons for flower coloration, a comprehensive comparison between metabolic compounds and transcriptome data was carried out to analyze the metabolite levels in different petal colors and the expression of the corresponding key genes of different branch pathways. The results showed that the pink petals of varying degrees might be influenced by several factors. For example, in the case of red and pink petals, firstly, Cy3G was the main pigment compound in PFS. The Cy metabolic pathway was dominant in the color formation-related metabolic system, and its metabolic product accounted for 77.43% of TFL metabolites, whereas in the pink petals, Cy metabolic products accounted for 22.49% of TFL metabolites. The Cy content in the red petals was 5.49 times higher than that in the pink petals (Fig. 5b). *FpF3’H* is the key gene regulating Cy metabolism, and relatively low productivity might restrict the lower production of the Cy branch in the red and pink petals, leadingd to the different shades of pink in PFS hybrids. Secondly, in the red petals, the content of Cy was 58.73 times and 229 times higher than Pgs and Dels content, respectively; In the pink petals, the content of Cy was 20.75 times and 155.78 times higher than Pgs and Dels content, respectively (Table 1). The substrate specificity of the *FpDFR* and *FpANS* genes might be the reason that anthocyanins in PFS were mainly Cy derivatives, thus leading to the red petals. Thirdly, the competition effect of the *FpFLS* gene also limited the biomass of Cy derivatives. As shown in Fig. 5, the total amount of flavonols produced in the pink petals accounted for 75.51% of the TFL contents, which was 2.85 times higher than that in red flowers. With the upregulated expression of the *FpFLS* gene, the amount of biomass that was originally used for the flow of Cy was turned into colorless flavonols. Ca was detected in PFS petals of different colors. The proportion of Ca content in TFL was similar to that in the red and pink petals, accounting for 2.54 and 2.26%, respectively, suggesting that the expression of the *FpLAR* gene in the red flower was maintained at a level similar to that in the pink flower. Therefore, PFS hybrids with different shades of pink might be due to the substrate specificity of the *FpDFR* and *FpANS* genes, with Cy derivatives as the main anthocyanin components. The expression of the *FpF3’H* and *FpFLS* genes was different, resulting in the production of different proportions of Cys and flavonols, thus affecting the color of flowers.

### Metabolism compounds associated with flower coloration in PFS

The petal color is one of the major characteristics that determine the ornamental value of PFS. Flavonoids widely exist in flowers, fruits and vegetables, such as anthocyanins, flavones and flavonols [28]. So far, several reports have shown the relationship between flower color and pigment composition and content [29–31]. One hundred and eight lotus cultivars with different colors were assessed through detection and quantification of pigments in their petals by Deng et al. (2013), who detected 5 anthocyanins and 14 flavones and flavonols, and the content and composition of these pigments varied considerably among different cultivars [32]. The red color of lotus petal was positively correlated with the anthocyanin content, especially with the content of delphinidin 3-O-glucoside and malvidin 3-O-glucoside. In this study, the anthocyanin content in the red petals was significantly higher than the flavonol content. Furthermore, the flavonol content was the highest in the white petal with no significant differences from that in light pink and pink petals, and no anthocyanin was detected in the white petals. The petal color changed from red to white, which was consistent with increased flavonol content and decreased anthocyanin content. Flavones and flavonols have also been reported to be responsible for the petal color [33]. However, the function was limited to flavonols, as a co-pigmentation, to change the petal color from magenta to blue color [34, 35]. Du et al. (2018) reported the anthocyanin composition of 30 *Rhododendron* species and showed that there was no significant difference in co-pigmentation index among color different groups and flavonols might not play a major role in coloration [36]. Our study results showed that the white petal was due to the significant accumulation of flavonols and that the key factor of shades of pink was the accumulation of anthocyanins. However, the influence of different proportions of anthocyanins and flavonols on the formation of different shades of pink petals in PFS needs further study. The metabolic regulation related to the formation of flower color is a complex metabolic pathway, which is regulated the key genes of several branch metabolic pathways and regulatory factors. It was difficult to obtain an absolute correlation between the genes and the corresponding metabolites. The anthocyanin composition and content and co-pigment flavonol content in the petals were analyzed to understand the biochemical basis of different flower colors and to predict the genetic differences.

### Mechanism of flower coloration in PFS

Some previous studies have provided sufficient evidence for the molecular mechanism of anthocyanin composition in strawberry fruits, but have not explained pigmentation in the petal of PFS [15, 37–39]. The main anthocyanin compounds mainly in the fruits and flowers of PFS are Pgs and Cys, respectively, which indicated that the ABP is regulated in a spatially controlled manner [21]. The ABP is regulated by complex transcription factors, which confer tissue specificity [40–42]. Matu et al. (2017) identified three closely related R2R3-MYB genes, namely, *MYBA5, MYBA6*, and *MYBA7*, located in chromosome 14 that controlled the ABP in vegetative organs with different specificities [43]. To date, different enzymes involved in the ABP of the strawberry fruit have been identified, such as several structural genes *FaCHS*, *FaF3H*, *FaDFR*, *FaANS*, *FaFLS*, and *FaGT1*, and some regulatory genes *FaMYB1*, *FcMYB1*, *FaMYB5*, *FaMYB10*, and *FabHLH33* [16, 17, 44]. Recently, Hawkins et al. (2016) demonstrated that a candidate SNP on chromosome 14 in *FveMYB10* was identified, and then functionally confirmed to be responsible for the yellow color fruits in several *F. vesca* accessions via genome-scale analyses [45]. In this study, 62 annotated unigenes encoding 40 MYB DEGs were annotated in our assembled transcriptome datasets, including the *MYB1*, *GAMYBA4*, *MYB6*, *MYB44*, *MYB82*, *MYB11*, *MYB305* and *MYB308* genes. However, only 10 recovered *MYB* genes were likely ABP-regulating genes, as inferred from their position within the phylogenetic tree, and most of those unigenes were transcriptional repressors (unpublished). It was different from the strawberry fruit, and the positive regulation gene *FaMYB10* was not annotated. Recently, more regulatory transcription factors have been identified by expression correlation analysis in strawberry fruit [46], indicating that our knowledge of the gene network of this pathway is still incomplete. All these data suggested the presence of additional regulators in anthocyanin synthesis in PFS. Furthermore, increasing evidences showed that various TF families, such as NAC, ARF, and WRKY, were involved in the regulation of ABP. In our study, a number of putative TF DEGs were selected, according to several TFs reported to play important roles in ABP. Therefore finding and identification some anthocyanin-related TFs in PFS might confirm that pigmentation is a complex trait possibly involving distinct regulation mechanism.

Further investigations into the enzymatic properties of the structural genes, their regulatory mechanisms, and the content of related metabolites are still required for a complete understanding of the ABP in PFS. In this study, more than one isoform was found for each structural gene via transcriptome data. This suggested that all the examined ABP genes in PFS would represent multigene families, which was also the case in several other species [47]. The Cy is the main anthocyanin in different flower colors of PFS, and its content was significantly higher than Pgs and Dels content (Table 1), which seemed to be caused by the higher *FpF3′H* gene expression than *FpF3H* and *FpF3′5′H* genes. Moreover, the substrate specificity of the *FpDFR*, *FpANS*, and *FpUGT* genes could contribute to widening the gap between the contents of Cys and other anthocyanins. The level of anthocyanin content was accounted for 79.1% of the TFL in the red petal, suggesting that the *FpDFR* gene has priority in the competition between *FpDFR* and *FpFLS* for their substrate dihydroflavonols. Similar results have been obtained in other horticultural plants [48, 49]. Park et al. (2017) reported that the Qr content was approximately 12-fold higher than the Cy content in red onion sheath, which confirmed that the *FpFLS* gene dominated the competition for their substrate dihydroquercetin [50]. The mechanism of different petal coloration in PFS was speculated and the reason for the white-flowered hybrid was lack of anthocyanins. The underlying mechanism is probably more complex than what is described here. The mechanism would be clarified by identifying characteristic structures of genes and by examining how the occurrence of transcription factors, transporters or miRNAs affected the flowers and fruits of PFS cultivars in our future work. Exploring the molecular mechanism of petal coloration might help apply genetic engineering to produce other novel colors of PFS.

## Conclusions

In this study, we explored the regulatory networks of the ABP in the petals of PFS at the metabolome and transcriptome levels. Overall, we identified a subset of candidate genes putatively associated with petal pigmentation including 57 flower coloration-related genes of ABP and 241 TFs. The mechanism of different petal coloration in PFS was speculated and the reason for the white-flowered hybrid was lack of anthocyanins. Our results make a significant contribution to the literature because it provides new insights for the subsequent study on complex physiological processes and molecular mechanisms associated with the biosynthesis and regulation of anthocyanins in the flowers of PFS.

## Methods

### Plant materials

**Fig. 6:**
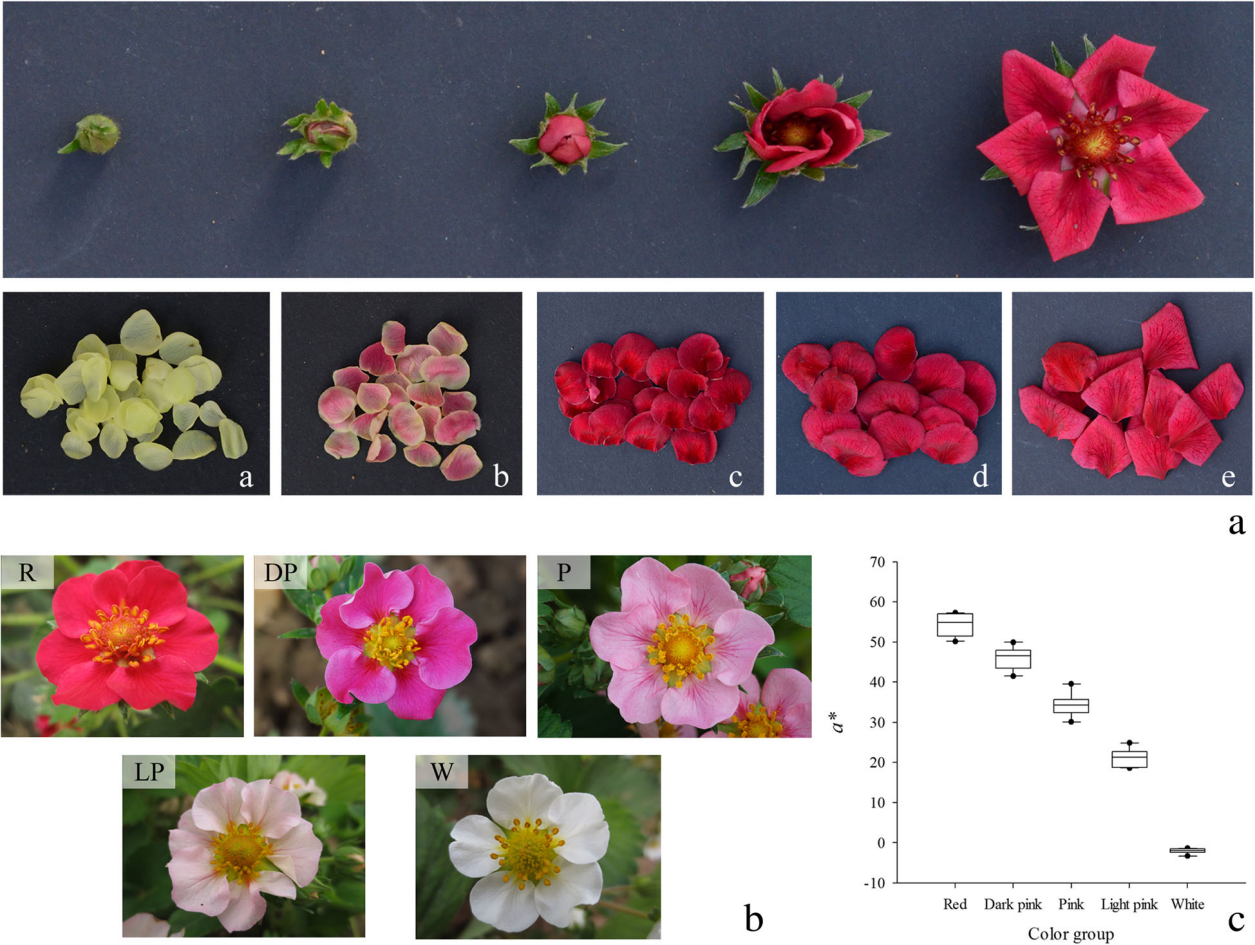
Materials chose for transcriptome sequencing and qRT-PCR analyses. A. The petal developmental stages of pink-flowered strawberry cultivar Sijihong. a, Young bud stage (L). b, Beginning coloration stage (Z). c, Big bud stage (D). d, Half opening stage (B). e, Full opening stage (S). The samples of ‘L’, ‘Z’ and ‘D’ were used for transcriptome sequencing with three biological replicates. B. Materials chose for qRT-PCR with different flower colors of hybrids from the cross of Pink Princess × Pretty Beauty. R. Red, DP. Dark pink, P. Pink, LP. Light pink, and W. White. C. a* value measured by a Chroma Meter (CR-400 Koniea Minolta, Japan) used to differentiate flower colors

The PFS cultivar Sijihong obtained from Pink Princess × Pretty Beauty with dark red flower. Its flower was divided into five developmental stages according to its opening degree and petal color (Fig. 6a, Table 3). The three developmental stages selected with significantly different flower colors (‘L’, ‘Z’ and ‘D’) were used for transcriptome analysis. The flower colors significantly varied among the hybrids obtained from the cross of Pink Princess × Pretty Beauty, ranging from white to red, and the hybrids were divided into the following five groups: red, dark pink, pink, light pink, and white (Fig. 6b). The fresh petal color of all hybrids and parents was measured using a Chroma Meter (CR-400 Koniea Minolta, Japan) (Fig. 6c, Additional file 1: Table S1). The fresh petals of cross hybrids from the five groups were sampled at full-bloom stage and used in the metabolome and qRT-PCR analyses. All samples were immediately frozen in liquid nitrogen and stored at − 80 °C for RNA extraction and flavonoid analysis. The samples of ‘L’, ‘Z’ and ‘D’ were used to construct nine libraries with three biological replicates, which were named as PH_L1, PH_L2, PH_L3, PH_Z1, PH_Z2, PH_Z3, PH_D1, PH_D2, and PH_D3, respectively.

**Table 3:** The characteristics of different developmental stages of the flower of pink-flowered strawberry cultivar Sijihong

Different developmental stages	Characteristics
Young bud stage (L)	Sepals are closed with colorless petals
Coloration beginning stage (Z)	Sepals are slightly open, closed petals appearing, and petals just begin coloration
Big bud stage (D)	Sepals are open, petals are also closed and fully colored
Half-opened stage (B)	Petals are partially open and longer than the sepals
Fully opened stage (S)	Sepals are fully spread and the flowers are fully opened

### Flavonoid determination in flowers

The anthocyanins in PFS petals were determined using HPLC-MS analysis as previously described [21]. Ten microliters of sample solution was injected for HPLC analysis. Anthocyanins were identified using an Agilent-1100 HPLC system coupled to an LC/MSD Trap SL electrospray ion mass spectrometer (Agilent 1100 LC/MSD Trap SL G2445A). The HPLC column used was DIKMA SpursilTMC18 (250 mm × 4.6 mm, 5 μm) and protected with a C18 micro-guard cation-H cartridge (10 mm × 4.6 mm; Shanghai ANPEL Scientific Instrument, Shanghai, China). The flow rate was 0.7 mL/min. The mobile phases A and B were composed of 0.1% (v/v) trifluoroacetic acid (TFA) in water and 0.1% (v/v) TFA in CH_3_CN, respectively. The column was eluted at 25 °C using the following linear gradients: 0 min, 100% A, 0%B; 40 min, 50% A, 50% B; 42 min, 100% A, 0% B; and 47 min, 100% A, 0% B. The MS condition was as follows: positive ion mode; gas (N_2_) temperature, 350 °C; flow rate, 12 L/min; nebulizer pressure, 35 psi; capillary voltage, − 4500 V; capillary exit, 200 V. Mass spectra were recorded from m/z 100 to 1000. Anthocyanins at 530 nm, flavones and flavonols at 350 nm were detected, and UV spectra were recorded between 200 and 800 nm. Data were recorded on a computer with Agilent Chemstation software (Chemsta-tion for LC 3D system REV. B. 01. 03). Cy3G, quercetin 3-O-rutinoside (Rutin), naringenin (Nrn), myricetin, Qr, Ka, dihydromyricetin (DHM), dihydroquercetin (DHQ), DHK, (+)-catechin (Ca), and (−)-epicatechin (Ep) were obtained from the National Institutes for Food and Drug Control (Beijing, China) and used as the standards for quantification. The flavonoids were identified based on the HPLC retention time, elution order, UV–vis spectrum, and MS fragmentation pattern by comparison with those of the standards and published data. The content of TA and TFL was calculated using the absorbance measured at 530 nm and 350 nm, respectively. The mean values and SEs were obtained from three biological replicates.

### RNA extraction, library construction, and sequencing

Total RNA was extracted using a modified CTAB method and treated with RNase-free DNase I (Takara, Dalian, China) to remove genomic DNA contamination. The total RNA quantity and purity were determined using Bioanalyzer 2100 and the RNA 6000 Nano LabChip Kit (Agilent, CA, USA) with RIN number of> 7.0. The methods of library construction and sequencing were the same to the published work [51]. In brief, Total RNA samples of 10 μg were extracted from each development stage. Then they were subjected to isolate poly (A) mRNA with poly-T oligo-attached magnetic beads. The cDNA library was constructed in accordance with the protocol of the mRNA-Seq Sample Preparation Kit (Illumina, San Diego, USA). The paired-end sequencing was at the lc-bio (China) using an Illumina Hiseq 4000 platform.

### De novo assembly, unigene annotation, and differential expression analysis

The raw data in FASTQ format have been deposited in the National Center for Biotechnology Information (NCBI) database (accession no. GSE125777). Cutadapt and perl scripts in house were used to remove the reads that contained adaptor contamination, low quality bases, and undetermined bases. The sequence quality was then verified using FastQC (http://www.bioinformatics.babraham.ac.uk/projects/fastqc/) including the Q20, Q30, and GC content of the clean data. All downstream analyses were based on clean data of high quality. The clean data of all the nine samples were combined for de novo assembly of the transcriptome with Trinity 2.4.0 [52]. Trinity groups transcripts into clusters based on shared sequence content. Such a transcript cluster was very loosely referred to as a “gene”. The longest transcript in the cluster was chosen as the “gene” sequence (i.e., unigene). All assembled unigenes were aligned against the non-redundant (Nr) protein database (http://www.ncbi.nlm.nih.gov/), GO (http://www.geneontology.org), SwissProt (http://www.expasy.ch/sprot/), KEGG (http://www.genome.jp/kegg/), and eggNOG (http://eggnogdb.embl.de/) databases using DIAMOND with a threshold of E-value < 0.00001 [53]. Differentially expressed unigenes were selected with |log2 (foldchange)| ≥1 and with statistical significance (FDR<0.05) using R package edgeR [54]. Next, the GO and KEGG enrichment analyses were repeated for the differentially expressed unigenes using the in house perl scripts, and significantly enriched GO terms and KEGG pathways were identified using hypergeometric tests with *p* ≤ 0.05 as a threshold [55, 56].

### Gene validation and expression analysis

The specific emphasis was placed on color-related secondary metabolism involved in flavonoid biosynthesis, including the phenylpropanoid biosynthetic pathway (ko00940), FBP (ko00941), and ABP (ko00942). All isoforms of each color-related gene present in the PFS transcriptome database examined were aligned using BLASTx. Some color-related key genes and transcription factors were chosen for validation in different flower colors of hybrids (red, dark pink, pink, and light pink) using qRT-PCR (Fig. 6). Three biological repeats were performed for each gene in each sample. Total RNA was extracted using a modified CTAB method and treated with RNase-free DNase I (Takara, Dalian, China) to remove genomic DNA contamination. The total RNA quantity and purity were determined using Bioanalyzer 2100 and the RNA 6000 Nano LabChip Kit (Agilent, CA, USA). The cDNA of each sample was synthesized using the PrimeScript RT reagent Kit (TaKaRa, Dalian, China). Primers were designed using the Primer 3.0 software (http://biotools.umassmed.edu/bioapps/primer3 www.cgi) for qRT-PCR and listed in Additional file 12: Table S6. The qRT-PCRs were performed on the IQ5 Real-Time Detection System (Bio-Rad, USA) with the SYBR Green PCR Master Mix (Takara, Dalian, China), and amplified with 1 μL of cDNA template, 5 μL of 2 × SYBR Green Master Mix, and 0.5 μL of each primer (10 μmol/μL), to a final volume of 10 μL by adding water. The amplification program consisted of one cycle at 95 °C for 3 min, followed by 40 cycles of 95 °C for 30 s and 60 °C for 30 s. The strawberry 26S rRNA gene was used as the reference gene. All the data were subjected to statistical analysis using Duncan’s multiple range test (SPSS ver.17.0), and they were indicated as mean ± SE.

## Supplementary information

**Additional file 1: Table S1.** The total anthocyanin and total flavonoid contents in the petals of three developmental stages (PF_L, PF_Z and PF_D) used for the RNA-seq analysis.

**Additional file 2: Table S2.** Overview of sequencing and assembly of the transcriptome.

**Additional file 3: Table S3.** N50 of transcripts or unigenes from nine samples.

**Additional file 4: Table S4.** Length of transcripts or unigenes from nine samples.

**Additional file 5: Figure S1.** Bioinformatic analysis of RNA-seq data. A, Pearson’s distance correlation matrix of gene expression in PF_L, PF_Z and PF_D. B, Venn diagram showing the overlap of differentially expressed genes between any two stages of the PFS petal. C, The number of differential expression genes between PF_Z and PF_L, PF_D and PF_L, and PF_D and PF_Z.

**Additional file 6: Figure S2.** Number and percentage of transcripts matching the eight top species using BLASTx in the Nr database.

**Additional file 7: Figure S3.** GO classification of DEGs in PF_L vs PF_Z vs PF_D. The biological process, cellular component and molecular function were analyzed.

**Additional file 8: Figure S4.** Correlation of gene expression obtained from qRT-PCR analysis and RNA-Seq for 17 color-related genes. All reactions of qRT-PCR were repeated three times for each sample, and vertical bars indicated standard errors. Red indicated the fold changes of transcript expression levels determined by qRT-PCR. Green indicated the fold changes generated from the high-throughput sequencing.

**Additional file 9: Figure S5.** Expression profile of flower color related genes was Z-score normalized and hierarchically clustered in the heatmap. A color scale is shown at the top. Blue color indicates lower expression, while red color indicates higher expression. L, Young bud stage; Z, Beginning coloration stage; D, Big bud stage.

**Additional file 10: Table S5.** The numbers of differential expressed TFs related with ABP in the three development stages.

**Additional file 11: Figure S6.** Expression profile of transcription factors related to anthocyanins biosynthesis was Z-score normalized and hierarchically clustered in the heatmap. A color scale is shown at the right. Green color indicates lower expression, while red color indicates higher expression. L, Young bud stage; Z, Beginning coloration stage; D, Big bud stage.

**Additional file 12: Table S6.** The primers used for qRT-PCR analysis.

## Data Availability

The sequencing data has been submitted to NCBI Gene Expression Omnibus (GEO) database (https://www.ncbi. nlm.nih.gov/geo/) under the accession number of GSE125777.

## Abbreviations

ABP: Anthocyanins biosynthesis pathway
Ca: Catechin
Cy: Cyanidin
Cy3G: Cyanidin 3-O-glucoside
DEG: Differentially Expressed Gene
Del: Delphinidin
DHK: Dihydrokaempferol
DHM: Dihydromyricetin
DHQ: Dihydroquercetin
Ep: Epicatechin
FBP: Flavonoid biosynthesis pathway
GO: Gene ontology
HPLC-MS: High-performance liquid chromatography coupled with mass spectrometry
Ka: Kaempferol
KEGG: Kyoto Encyclopedia of Genes and Genomes
NCBI: National Center for Biotechnology Information
NJ: neighbour joining
Nr: non-redundant protein database
Nrn: Naringenin
PFS: Pink-flowered strawberry
Pg: Pelargonidin
Qr: Quercetin
Rutin: Quercetin 3-O-rutinoside
TA: Total anthocyanins
TFA: Trifluoroacetic acid
TFL: Total flavonoids
