# Supplementary material for: Study on cyanidin metabolism in petals of pink-flowered strawberry based on transcriptome sequencing and metabolite analysis
Source: BMC Plant Biol. 2019 Oct 14;19:423. doi: 10.1186/s12870-019-2048-8 (PMC6791029; doi:10.1186/s12870-019-2048-8)
Supplement: Supplementary file 1 — Additional file 1: Table S1. The total anthocyanin and total flavonoid contents in the petals of three developmental stages (PF_L, PF_Z and PF_D) used for the RNA-seq analysis. [file 12870_2019_2048_MOESM1_ESM.doc]

| Table S1 The total anthocyanin and total flavonoid contents in the petals of three developmental stages (PF_L, PF_Z and PF_D) used for the RNA-seq analysis | | | | | |
| --- | --- | --- | --- | --- | --- |
| Samples | *l** | *a** | *b** | TA | TFL |
| PF_L | 63.25±0.99 | -10.89±0.56 | 36.82±0.81 | — | 361.16 ±5.56 |
| PF_Z | 59.22±0.24 | -7.72±0.13 | 34.15±0.26 | 191.68±8.66 | 827.66±13.17 |
| PF_D | 39.60±0.31 | 49.61±1.23 | 17.47±0.13 | 1670.02±17.96 | 2344.36±22.36 |
| Note: The samples of ‘L’, ‘Z’ and ‘D’ were used for transcriptome sequencing; *l**, lightness; *a** and *b**, chromatic components; TA, Total anthocyanins; TFL, Total flavonoids. | | | | | |
