# Supplementary material for: Study on cyanidin metabolism in petals of pink-flowered strawberry based on transcriptome sequencing and metabolite analysis
Source: BMC Plant Biol. 2019 Oct 14;19:423. doi: 10.1186/s12870-019-2048-8 (PMC6791029; doi:10.1186/s12870-019-2048-8)
Supplement: Supplementary file 2 — Additional file 2: Table S2. Overview of sequencing and assembly of the transcriptome. [file 12870_2019_2048_MOESM2_ESM.doc]

| Table S2 Overview of sequencing and assembly of the transcriptome | | | | | | | | |
| --- | --- | --- | --- | --- | --- | --- | --- | --- |
| Samples | Raw_Reads | Raw_Bases | Valid_Reads | Valid_Bases | Valid% | Q20% | Q30% | GC% |
| PF_L1 | 38,549,916 | 5.82G | 37,860,886 | 5.59G | 98.21 | 98.34 | 95.38 | 46.71 |
| PF_L2 | 55,718,164 | 8.41G | 54,680,870 | 8.08G | 98.14 | 98.33 | 95.39 | 46.93 |
| PF_L3 | 49,721,048 | 7.51G | 48,815,484 | 7.21G | 98.18 | 98.29 | 95.28 | 46.77 |
| PF_Z1 | 53,556,478 | 8.09G | 52,576,062 | 7.76G | 98.17 | 98.21 | 95.19 | 46.83 |
| PF_Z2 | 45,358,060 | 6.85G | 43,731,870 | 6.47G | 96.41 | 98.35 | 95.51 | 46.91 |
| PF_Z3 | 54,078,570 | 8.17G | 53,117,628 | 7.86G | 98.22 | 98.41 | 95.53 | 46.79 |
| PF_D1 | 56,766,470 | 8.57G | 54,757,060 | 8.10G | 96.46 | 98.33 | 95.42 | 46.94 |
| PF_D2 | 56,353,562 | 8.51G | 55,152,082 | 8.15G | 97.87 | 98.30 | 95.33 | 46.39 |
| PF_D3 | 53,828,940 | 8.13G | 52,650,558 | 7.78G | 97.81 | 98.34 | 95.39 | 46.52 |
