# Supplementary material for: Study on cyanidin metabolism in petals of pink-flowered strawberry based on transcriptome sequencing and metabolite analysis
Source: BMC Plant Biol. 2019 Oct 14;19:423. doi: 10.1186/s12870-019-2048-8 (PMC6791029; doi:10.1186/s12870-019-2048-8)
Supplement: Supplementary file 3 — Additional file 3: Table S3. N50 of transcripts or unigenes from nine samples. [file 12870_2019_2048_MOESM3_ESM.doc]

| Table S3 N50 of transcripts or unigenes from nine samples | | | | | | | |
| --- | --- | --- | --- | --- | --- | --- | --- |
| Index | All | GC% | Min Length | Median Length | Max Length | Total Assembled Bases | N50 |
| Transcript | 164,727 | 42.55 | 201 | 606 | 12583 | 141,249,559 | 1,250 |
| Gene | 50,285 | 42.47 | 201 | 548 | 12583 | 43,531,127 | 1,394 |
