# Supplementary material for: Study on cyanidin metabolism in petals of pink-flowered strawberry based on transcriptome sequencing and metabolite analysis
Source: BMC Plant Biol. 2019 Oct 14;19:423. doi: 10.1186/s12870-019-2048-8 (PMC6791029; doi:10.1186/s12870-019-2048-8)
Supplement: Supplementary file 4 — Additional file 4: Table S4. Length of transcripts or unigenes from nine samples. [file 12870_2019_2048_MOESM4_ESM.doc]

| Table S4 Length of transcripts or unigenes from nine samples | | | | | |
| --- | --- | --- | --- | --- | --- |
|  | 200 - 500 bp | 500 -1kbp | 1k – 2 kbp | > 2 kbp | Total |
| Number of transcripts | 69 579 | 45 460 | 36 935 | 12 753 | 164 727 |
| Number of unigenes | 23 669 | 11 143 | 10 846 | 4 627 | 50 285 |
