# Supplementary figures and images for: Study on cyanidin metabolism in petals of pink-flowered strawberry based on transcriptome sequencing and metabolite analysis
Source: BMC Plant Biol. 2019 Oct 14;19:423. doi: 10.1186/s12870-019-2048-8 (PMC6791029; doi:10.1186/s12870-019-2048-8)

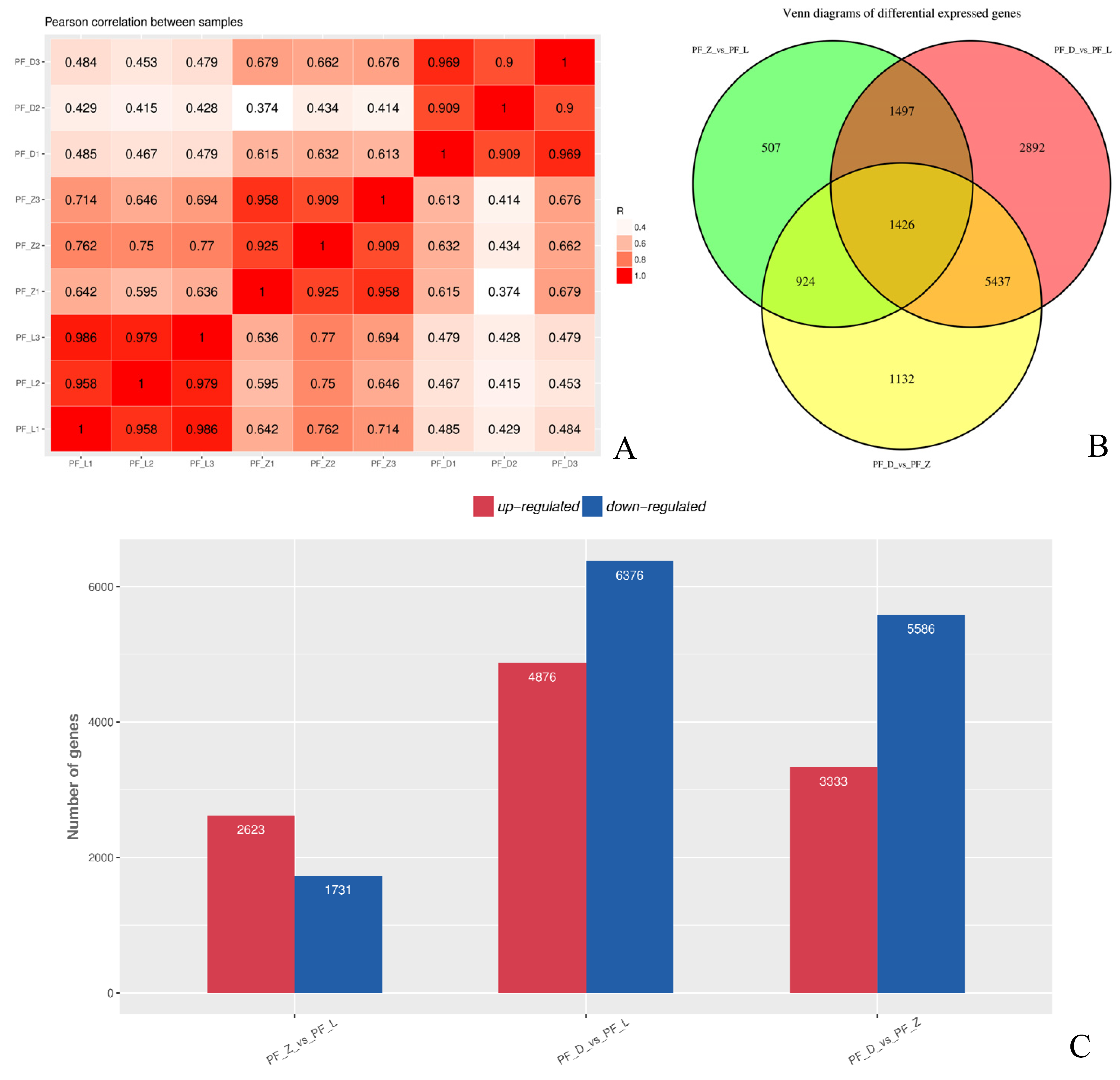

Supplement: Supplementary file 5 — Additional file 5: Figure S1. Bioinformatic analysis of RNA-seq data. A, Pearson’s distance correlation matrix of gene expression in PF_L, PF_Z and PF_D. B, Venn diagram showing the overlap of differentially expressed genes between any two stages of the PFS petal. C, The number of differential expression genes between PF_Z and PF_L, PF_D and PF_L, and PF_D and PF_Z. [file 12870_2019_2048_MOESM5_ESM.tif]

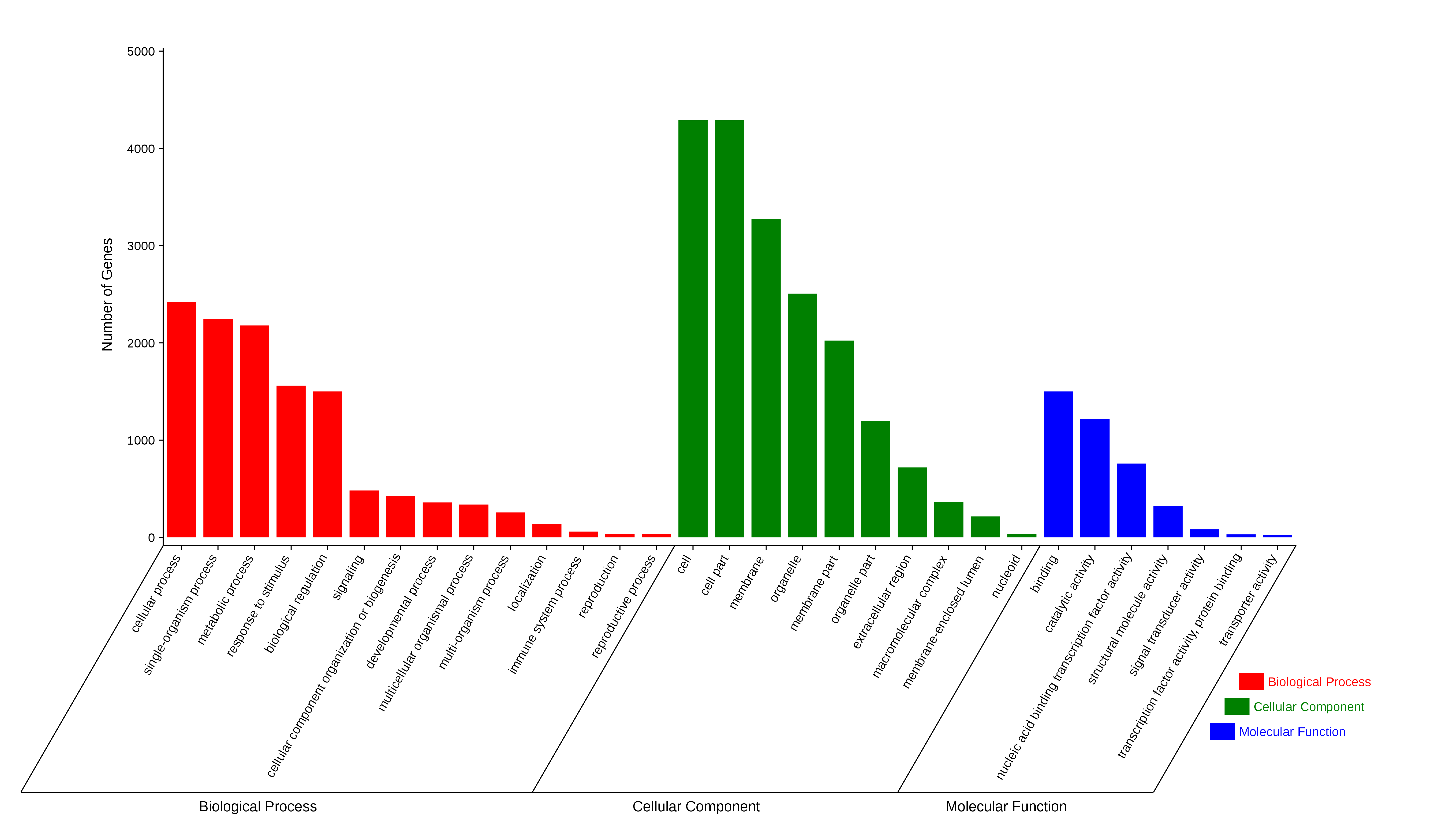

Supplement: Supplementary file 7 — Additional file 7: Figure S3. GO classification of DEGs in PF_L vs PF_Z vs PF_D. The biological process, cellular component and molecular function were analyzed. [file 12870_2019_2048_MOESM7_ESM.tif]

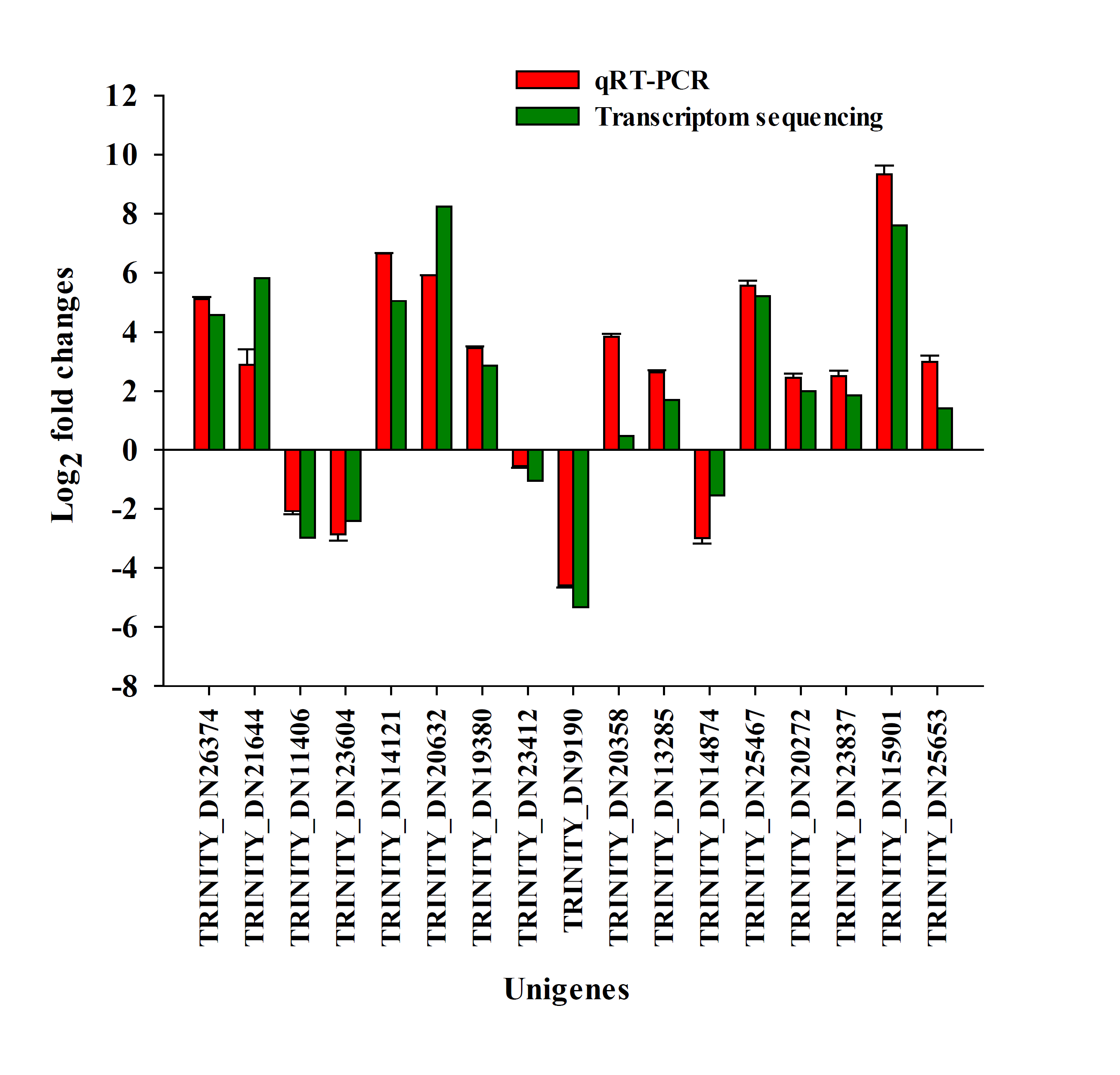

Supplement: Supplementary file 8 — Additional file 8: Figure S4. Correlation of gene expression obtained from qRT-PCR analysis and RNA-Seq for 17 color-related genes. All reactions of qRT-PCR were repeated three times for each sample, and vertical bars indicated standard errors. Red indicated the fold changes of transcript expression levels determined by qRT-PCR. Green indicated the fold changes generated from the high-throughput sequencing. [file 12870_2019_2048_MOESM8_ESM.tif]

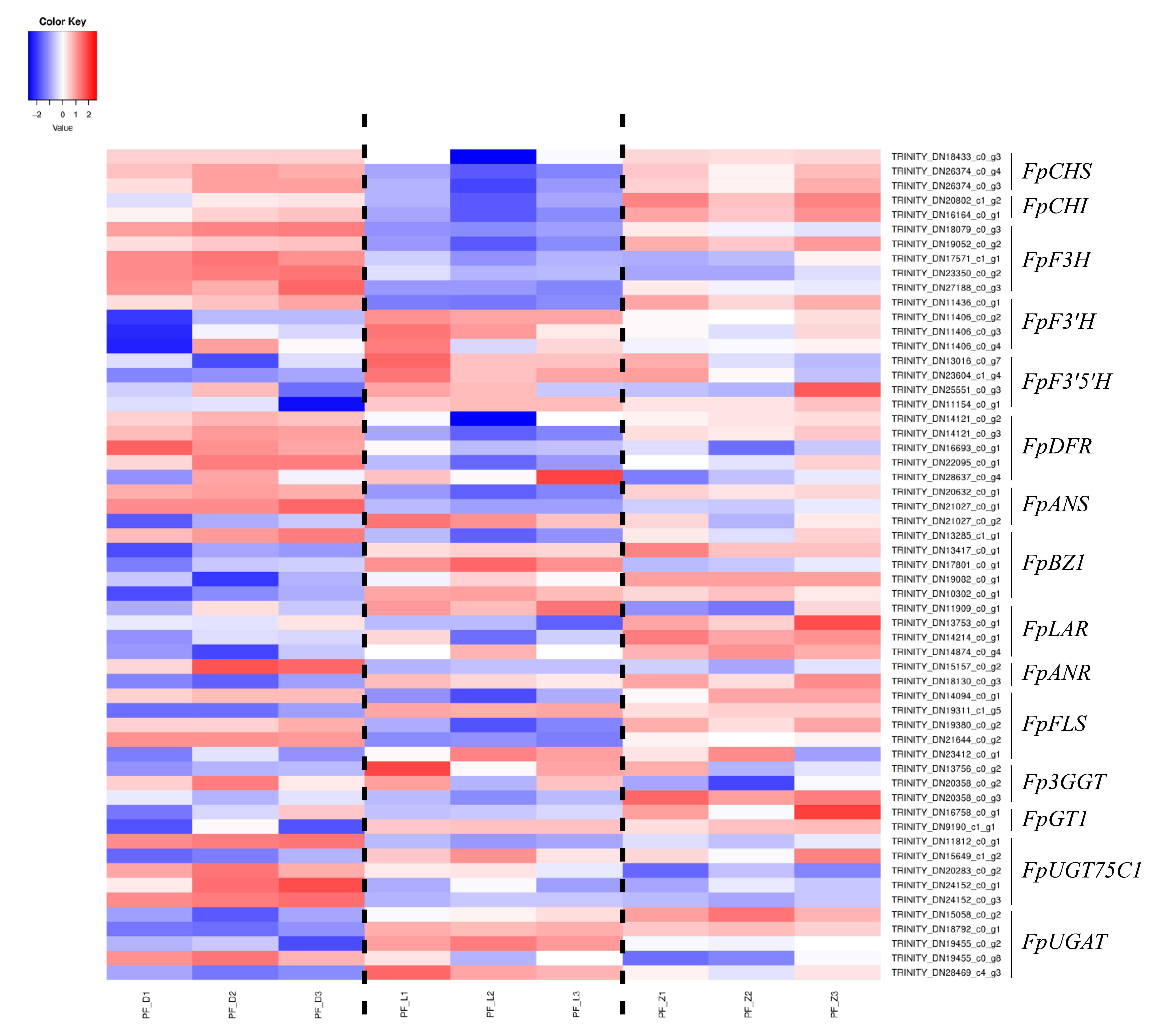

Supplement: Supplementary file 9 — Additional file 9: Figure S5. Expression profile of flower color related genes was Z-score normalized and hierarchically clustered in the heatmap. A color scale is shown at the top. Blue color indicates lower expression, while red color indicates higher expression. L, Young bud stage; Z, Beginning coloration stage; D, Big bud stage. [file 12870_2019_2048_MOESM9_ESM.tif]

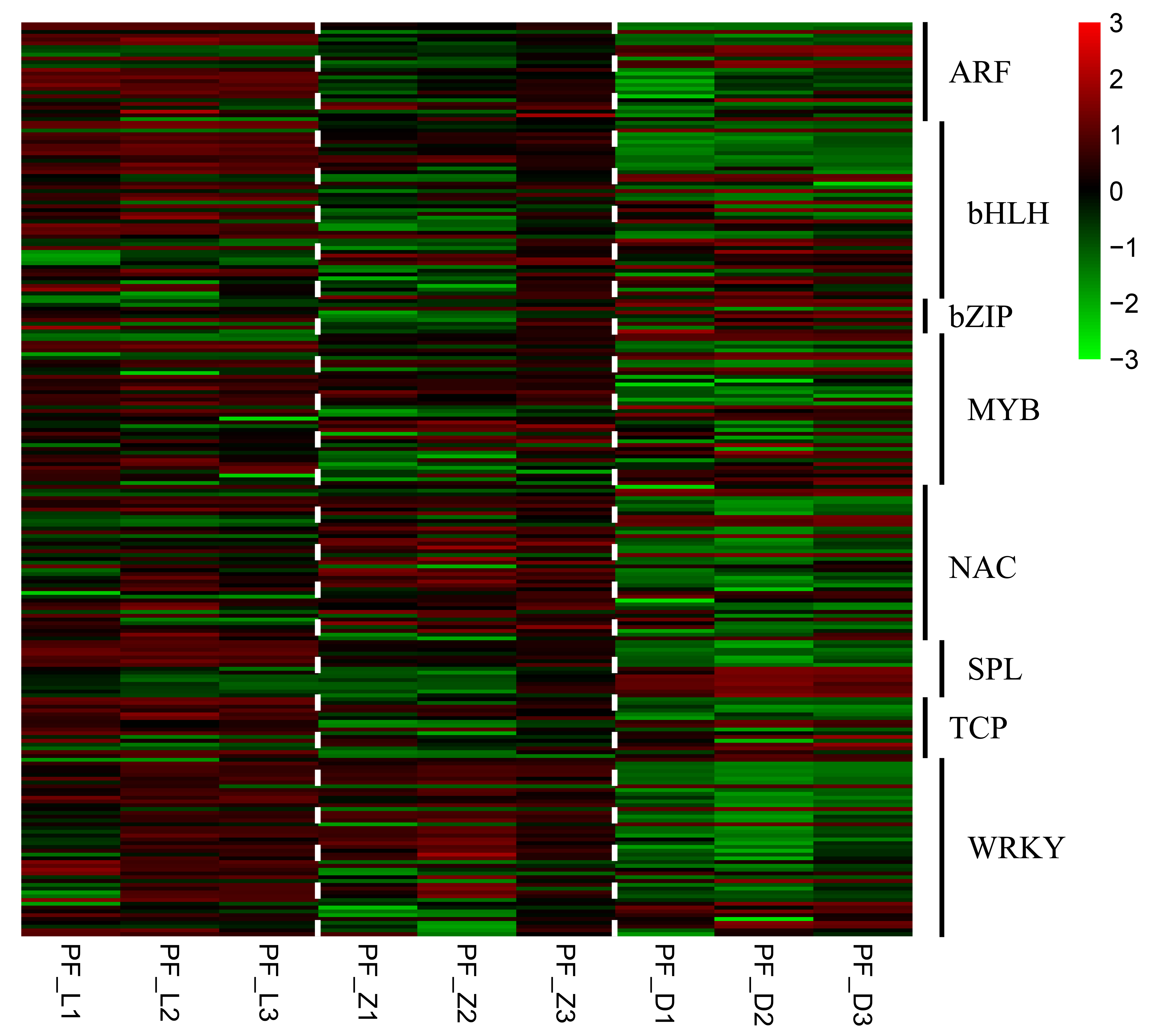

Supplement: Supplementary file 11 — Additional file 11: Figure S6. Expression profile of transcription factors related to anthocyanins biosynthesis was Z-score normalized and hierarchically clustered in the heatmap. A color scale is shown at the right. Green color indicates lower expression, while red color indicates higher expression. L, Young bud stage; Z, Beginning coloration stage; D, Big bud stage. [file 12870_2019_2048_MOESM11_ESM.tif]
