# Supplementary material for: Study on cyanidin metabolism in petals of pink-flowered strawberry based on transcriptome sequencing and metabolite analysis
Source: BMC Plant Biol. 2019 Oct 14;19:423. doi: 10.1186/s12870-019-2048-8 (PMC6791029; doi:10.1186/s12870-019-2048-8)
Supplement: Supplementary file 12 — Additional file 12: Table S6. The primers used for qRT-PCR analysis. [file 12870_2019_2048_MOESM12_ESM.doc]

| Table S6 The primers used for qRT-PCR analysis | | | |
| --- | --- | --- | --- |
| Genes | Description | Sequences（5' - 3'） | Amplicons（bp） |
| *FpCHS* | Forward | GCCTGAGAAGTTAGAAGCCACG | 161 |
|  | Reverse | CGAACCCAAACAGAACACCC |  |
| *FpCHI* | Forward | AGCATCACCCTCTACCCTCAT | 105 |
|  | Reverse | ACCCACTGCACCCATAGCTG |  |
| *FpF3H* | Forward | TGTGGCGTTTGAGTCCGAGA | 170 |
|  | Reverse | TGACGAGCTGATGGGGTTGG |  |
| *FpF3'H* | Forward | TCCTTGACTCGCTGCCTTGT | 204 |
|  | Reverse | CGTTCGGTGATGAAGCTCGA |  |
| *FpF3'5'H* | Forward | TCCGAGATGTTTGGTTTTGCGA | 104 |
|  | Reverse | TGGGGTGCCACTCTACAAGG |  |
| *FpDFR* | Forward | GCCTTCCACAGCGTCAAGTG | 142 |
|  | Reverse | TCGTCGGATCATGGCTGGTC |  |
| *FpANS* | Forward | ATCTTCTCCTTGGGCGGCTC | 206 |
|  | Reverse | AACATGGTTCCCGGTCTGCA |  |
| *Fp BZ1* | Forward | AACTTGGGCTCCTCAGTCGG | 223 |
|  | Reverse | CTCCCTCCCTGCCACTTGTT |  |
| *FpBZ2* | Forward | AAAGGAGCTGATGGACCTCG | 104 |
|  | Reverse | ATGAAGCATATGAGGAACCCC |  |
| *FpBZ3* | Forward | GGCAACCAGTTTTCCTCGAT | 102 |
|  | Reverse | TGATGCCCAGCTCCTAGAAA |  |
| *FpUFGT75C1* | Forward | AGCCACAAGTCCCACGTCAA | 111 |
|  | Reverse | GTCGATCATCCCCGTGCAGA |  |
| *FpGT1* | Forward | AGACCCCACGCTAGTTCTTCA | 107 |
|  | Reverse | TGCATGAAGTGGCTAGACTCGA |  |
| *FpUFGT75C2* | Forward | TACGAGGTATGGATTGGTTGGC | 80 |
|  | Reverse | TCTTCACCTCACATCTTGCACC |  |
| *FpUGT79B1* | Forward | GCTGTTTGACCTCTTTGAGCGA | 108 |
|  | Reverse | TGACTGGCTTGTTCATCTGCA |  |
| *FpANR* | Forward | ATAGGTTCAACTCCGACCGC | 164 |
|  | Reverse | CCCGTTGAAGGTCCGAATGA |  |
| *FpLAR* | Forward | TTCTCAATCGCACGCCTCAC | 91 |
|  | Reverse | AGTTCGGGCATGACGTGGA |  |
| *FpFLS* | Forward | AGCTCCAATATCTTGCCCTCCA | 103 |
|  | Reverse | AGTAGTCAAGCTGTGTGGGAGA |  |
| *FpMYB1* | Forward | AGGGTTGCGTCGTTGTGGTA | 164 |
|  | Reverse | TTCCAGGCAGTCTTCCAGCT |  |
| *FpGAMYB* | Forward | ACTTGCGATTGAGACTTGTGGT | 125 |
|  | Reverse | CAGGAAGCAATCGTATACCCCG |  |
| *FpMYB11* | Forward | TGGCCGAACAGACAATGAAATCA | 117 |
|  | Reverse | TGATTTGGTGGTTTGTGGTGAGT |  |
| *FpbHLH77* | Forward | GGTAACTGGGAAAGCTGTGATGC | 100 |
|  | Reverse | ATTCACAGTCGCCAATTTCATCG |  |
| *FpbHLH79* | Forward | TCGGGCAGTTTGGGGATGTT | 153 |
|  | Reverse | AATCACCCGCGCCAATGTTG |  |
| *FpbHLH148* | Forward | GATCAAGTTCCGGCAGCACC | 111 |
|  | Reverse | AACCCCTTCCCCTTCAACCG |  |
| *Fp26S* | Forward | TAACCGCATCAGGTCTCCAA | 164 |
|  | Reverse | CTCGAGCAGTTCTCCGACAG |  |
